# Habitual Running Style Matters: Duty Factor, and Not Stride Frequency, Relates to Loading Magnitude

**DOI:** 10.5114/jhk/191528

**Published:** 2024-09-26

**Authors:** Lennert Van der Meulen, Senne Bonnaerens, Ine Van Caekenberghe, Dirk De Clercq, Veerle Segers, Pieter Fiers

**Affiliations:** 1 Department of Movement and Sports Sciences, Ghent University, Ghent, Belgium.; 2 Department of Movement and Sports Sciences, Vrije Universiteit Brussel, Brussels, Belgium.

**Keywords:** inverse dynamics, musculoskeletal load, external forces

## Abstract

Running style is temporally defined by a duty factor and stride frequency and believed to be related to the loading experienced during ever step. However, the exact relationship between both temporal variables and loading magnitude is still unknown. We aimed to identify the relationship between a duty factor and stride frequency with external load measures, joint reaction forces and joint moments. Thirty-one healthy female recreational runners ran across a 25-m runway at a speed of 2.30 ± 0.05 m·s^−1^. Ground reaction forces and motion capture data were used to determine the maximal vertical ground reaction force, the vertical instantaneous loading rate, peak braking force, peak joint extension moments and peak joint reaction forces at the knee and the ankle. The habitual duty factor and stride frequency of runners did not correlate with each other. The duty factor was found to be a significant predictor of maximal vertical ground reaction force (R^2^ = 0.585), peak braking force (R^2^ = 0.153), peak knee extension moment (R^2^ = 0.149), ankle plantar flexion moment (R^2^ = 0.225) and peak joint reaction forces at the knee (R^2^ = 0.591) and the ankle (R^2^ = 0.592), but not of the vertical instantaneous loading rate. Stride frequency had no significant predictive value. In conclusion, the maximal loading and potential injury risk of female recreational runners running with high duty factors are lower compared to those of peers running with lower duty factors.

## Introduction

Running is one of the most popular leisure activities due to its low cost, easy accessibility (Oja et al., 2015) and associated health benefits (Lee et al., 2017; Wen et al., 2011). Most of the runners predominantly run for short (5 km) and intermediate (10–21 km) distances in a recreational manner. Despite its preventive effect on the development of chronic diseases and all-cause mortality (Pedisic et al., 2020a), running has a major drawback, i.e., running-related injuries (RRIs). Within a time-period of one year, 46% of recreational runners develop a RRI (Jungmalm et al., 2018; Kluitenberg et al., 2015). These injuries are amongst the most important reasons why people stop running, preventing the continuation of a healthy and active lifestyle (Videbæk et al., 2015).

Within the etiology of RRIs, the magnitude of the load exerted on a tissue predominantly determines whether the tissue becomes damaged and the runner gets injured (Edwards, 2018). External ground reaction forces are used as an operational measure for the experienced loading and are determined by the runner’s speed and running style (Edwards et al., 2008; Morin et al., 2007a; Napier et al., 2019). Therefore, the running style is often examined in relation to RRIs (Benca et al., 2020; Ceyssens et al., 2019; Malisoux et al., 2022). The habitual running style can be defined by the use of the conceptual dual-axis framework proposed by van Oeveren and colleagues (2021). According to this framework, an individual’s running style can be defined by stride frequency (SF) and the duty factor (DF) when running at a constant speed. Based on those variables, five different habitual running styles are identified among recreational runners: ‘bounce’, ‘stick’, ‘push’, ‘sit’ and ‘hop’. Focusing on the running style of recreational runners, a large variation in both DF and SF can be observed with the DF ranging from 42.5 to 56.5% and SF ranging from 1.1 to 1.7 strides·s^−1^ when running at the same speed (2.1–2.6 m/s) (Bonnaerens et al., 2021; van Oeveren et al., 2021).

Focusing on the relationship between the running style and the experienced load, the two mass model of Clark and colleagues (2017) predominantly relates the magnitude of loads associated with spring-mass dynamics to the DF, whereas impact loading is more closely related to SF. Indeed, Bonnaerens and colleagues (2021) found that 75.5% of the variance in maximal vertical ground reaction force (FzMax) and 43.0% of the variance in peak braking force (PBF) could be explained by the DF, whereas changes in load measures such as vertical and antero-posterior ground reaction forces at the initial stance and peak tibial impact accelerations tend to be more associated with changes in SF (Clarke et al., 1985; Crowell and Davis, 2011; Lieberman et al., 2015). As previous research focused on the relationship between the running style and loading magnitude based on external ground reaction forces, it is unknown how a running style relates to more detailed internal load measures derived from inverse dynamics such as joint moments and joint reaction forces.

The aim of this study was twofold. First, we attempted to identify whether the habitual SF was related to the habitual DF of an individual. Based on observational data, it was expected that these temporal variables would be not or only weakly related (Bonnaerens et al., 2021; Patoz et al., 2020). Second, the study determined the relationship between the habitual running style and external and internal load measures. It was hypothesized that recreational runners who ran with higher DFs, would experience lower FzMaxs, PBFs, joint moments and joint contact forces, while recreational runners who ran with higher SFs would experience lower vertical instantaneous loading rates (VILRs). In this study, we purposefully shifted our focus from conventional athletic study subjects, who tend to exhibit lower susceptibility to running-related injuries (RRI), to slower female recreational runners. These individuals, often characterized by older age, a slightly slower running pace, a higher BMI, and a substantial representation in distance running events, present an important and underexplored cohort for investigation (Shorten and Pisciotta, 2017).

## Methods

### Participants

Fifty-nine female, recreational slow runners between the age of 35 and 55 participated in the experiment (age: 43 ± 5 year, body mass: 65.6 ± 6.8 kg, body height: 1.66 ± 0.06 m). Participants were recruited based on a questionnaire inquiring running habits and were included if they reported an average running speed below or equal to 2.64 m·s^−1^, ran more than 3 km/week and less than 30 km/week and did not suffer from a RRI three months prior to the experiment. The experimental design was approved by the ethics committee of the Ghent University Hospital (protocol code: BC-08373; approval date: 14 September 2020) and written informed consent was obtained from all participants before the start of the study.

### Measures

Prior to the experiment, anthropometrical data were measured and a set of 40 retroflective markers were placed on the lower body and hips. Three dimensional full body kinematic recordings were collected using a 12 Oqus camera motion capture system (Qualisys AB, Gothenburg, Sweden) measuring at 500 Hz. Ground reaction forces (GRFs) were simultaneously recorded at 1000 Hz using three consecutive force plates embedded in the runway (AMTI: 46 x 207 cm; Kistler: 60 x 40 cm, AMTI: 120 x 120 cm). Both systems were synchronized automatically by the Qualisys track manager software. Running speed was measured by a digital distance laser at 1000 Hz (NOPTEL: CMP3-30) and used to guide participants to the target running speed and check for constant running speed.

### Design and Procedures

After a 5-min warm-up, participants ran continuously up and down a 25-m runway at a speed of 2.30 m·s^−1^ (i.e., the median running speed of the recruited population) until three successful running bouts were recorded. Running bouts were considered successful if the running speed was constant, if the average running speed was within ± 0.05 m·s^−1^ of the target speed and if foot contacts were fully placed on one of the three force plates without targeting.

### Data Reduction and Analysis

Only strides of the right leg were processed. For every stride taken in the three running bouts, the DF was determined. After elimination of outliers (z-score greater than 3 or lower than −3), the median of the DF was selected as a representative stride for this participant. Thirty-one out of 59 runners were retained for further analysis to ensure uniform distribution in the DF across the test sample. As such, we avoided the potential influence of a larger number of samples clustered around a single duty factor and stride frequency on the comprehensive relationship with loading metrics. Anthropometric and temporal characteristics of participants retained for further analysis were not different from the entire test population (Table 1).

**Table 1:** Anthropometrical measurements and temporal characteristics of participants habitual running style

	All participants (n = 59)	Retained for analysis (n = 31)
BMI (kg·m^−2^)	23.92 (19.99–29.18)	23.57 (19.99–28.66)
Body mass (kg)	65.6 (49.9–79.0)	65.1 (49.9–78.6)
Stature (m)	1. 66 (1.48–1.79)	1.66 (1.48–1.76)
Fat content (%)	32.68 (15.45–45.70)	31.75 (21.15–43.00)
Duty factor (%)	41.25 (35.60–46.67)	41.09 (37.63–45.43)
Stride frequency (strides·s^−1^)	1.36 (1.18–1.48)	1.37 (1.24–1.46)

Temporal variables were derived from gait events based on vertical GRFs. The initial contact and the toe-off were defined as the instant the vertical GRF rose above and dropped below 70 N. Contact time was defined as the time between the initial contact and the toe-off, stride time as the time between two consecutive initial contacts of the same foot and SF as the inverse of stride time. The DF was obtained by dividing contact time by stride time.

The external load refers to load measures derived from external ground reaction forces without consideration of kinematics. GRFs used to calculate FzMax and PBF were low-pass filtered at 30 Hz using a second-order zero-lag Butterworth filter. FzMax and PBF were defined as the maximal vertical and the minimal antero-posterior GRF during contact, respectively. For determination of the maximal VILR, vertical GRFs were lowpass filtered at 50 Hz using a second order zero-lag Butterworth filter. The VILR was defined as the maximum of the time derivative of the vertical GRF during the first 50 ms of contact.

Internal load measures were calculated using inverse dynamics. Visual 3D software (C-motion, Germantown, MD) was used to create a model with the following segments: a foot, a shank, a thigh and a pelvis. Individual segments’ pose estimation was done using a 6 DOF algorithm. The peak knee extension moment and the plantar ankle flexion moment were defined as the maximal knee extension and plantar ankle flexion moment during contact. The peak joint reaction forces at the knee and the ankle were defined as the maximal joint reaction force at the knee and the ankle during the stance. Measures were normalized to the percentage of body weight. For inverse dynamics’ calculations, marker data and ground reaction forces were lowpass filtered using a second-order zero-lag Butterworth filter with a cut-off frequency of 15 Hz.

### Statistical Analysis

Statistical analyses were performed using SPSS (version 27.0). Descriptive analysis identified no outliers. Outliers were defined as values with a z-score greater than 3 or lower than −3. In order to identify if the DF and/or SF (independent variables) could predict the load measures (dependent variables), a multiple linear regression model using a step-wise selection procedure was calculated for all load metrics separately. Exclusion criteria were set at an F-value with *p* > 0.1 The level of significance was set at *p* < 0.05.

## Results

The DF and SF varied between participants from 37.63% to 45.43% and from 1.24 to 1.46 strides·s^−1^ (74.4 to 87.6 strides•min^−1^), respectively. The DF did not correlate with SF (r = 0.036, *p* = 0.846). The DF and SF both correlated with contact time (DF: r = 0.791, *p* < 0.001; SF: r = −0.514, *p* = 0.003 ) and swing time (DF: r = −0.763, *p* < 0.01, SF: r = −0.636, *p* < 0.01).

**Figure 1:**
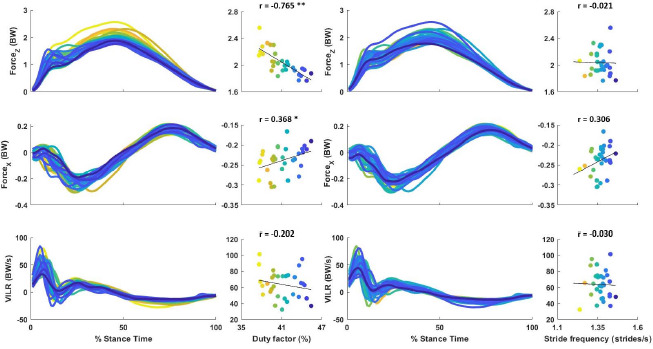
Correlation of the DF and SF with external load measures of FzMax, PBF and the VILR at a running speed of 2.3 m·s^−1^ in 31 female recreational runners. * significantly different at p ≤ 0.05, ** significantly different at p ≤ 0.01, FzMax: maximal vertical ground reaction force, PBF: peak braking force, VILR: vertical instantaneous loading rate

Figure 1 depicts the correlations (univariate r values are also presented) between the independent variables DF and SF and the external load measures. The DF was found to be a significant predictor of FzMax (R^2^ = 0.585, *p* < 0.001, B = −0.061) and PBF (R^2^ = 0.153, *p* = 0.042, B = 0.005). SF was excluded in both models as it did not improve the prediction of FzMax and PBF. For the VILR, neither the DF nor SF could be entered into the model.

**Figure 2:**
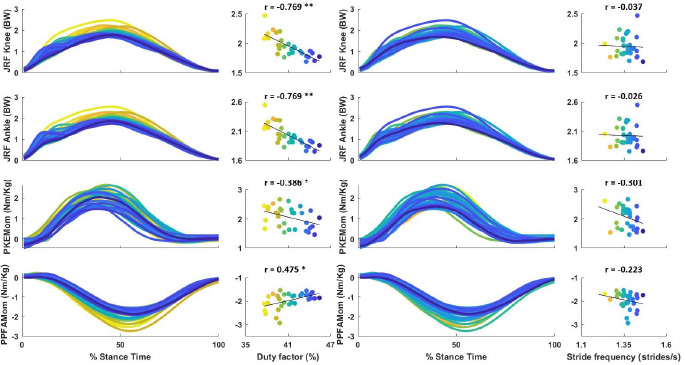
Correlation of the DF and SF with internal load measures of PPFAMom, PKEMom, JRFKnee and JRFAnkle at a running speed of 2.3 m·s^−1^ in 31 female recreational runners. * significantly different at p ≤ 0.05, ** significantly different at p ≤ *0.01, PPFAMom: peak plantar flexion ankle moment, PKEMom: peak knee extension moment, JRFAnkle: peak joint reaction force at the ankle, JRFKnee: peak joint reaction force at the knee*

The DF was found to be a significant predictor of all internal load measures, while SF was excluded from all models (peak plantar flexion ankle moment (R^2^ = 0.225, *p* = 0.007, B = 0.069), peak knee extension moment (R^2^ = 0.149, *p* = 0.032, B= −0.059), peak knee reaction force (R^2^ = 0.591, *p* < 0.001, B = −0.059) and peak ankle reaction force (R^2^=0.592, *p* < 0.001, B = −0.061)).

## Discussion

Our study investigated the relationship between the DF and SF as well as the relationship of those variables with internal and external loading magnitude. Overall, our study showed that in the habitual running pattern of slow, female runners, SF and the DF were not related. Furthermore, the DF related to lower FzMaxs, PBFs, peak joint moments and peak joint reaction forces at the knee and the ankle, while SF did not.

Recreational runners show a large interindividual variation in the DF and SF when running at the same slow speed that is close to their habitual running speed (the median running speed of the recruited population). Similarly to the results reported by Bonnaerens and colleagues (2021), the DF and SF were found to be independent from each other. Therefore, the magnitude of the experienced musculoskeletal load might be determined by none, one or both of these temporal variables when running at slow speeds.

At the same, slow speed, the DF was found to correlate significantly with FzMax and PBF, while SF did not correlate with any external measure. This confirms the results of Bonnaerens and colleagues (2021), stating that the DF is the strongest predictor of FzMax and PBF. Mechanically, running can be modelled as a spring-mass system with massless springs, the legs, connected to a point mass, the body center of mass (spring-mass system) (Blickhan, 1989; Farley and Fer, 1998; McMahon and Cheng, 1990). The relationship between FzMax and the DF according to the spring-mass dynamics is defined by:


$$FzMax=\frac{m.g.\pi .DF^{-1}}{4}$$

where FzMax is the maximal vertical ground reaction force, m is the mass of the runner, g is the gravitational constant and DF is the duty factor. According to this model’s dynamics, the DF is the major determinant of the external force magnitude. Our results confirm the theoretical approach indicating a clear inverse relationship of FzMax with the DF. To the best of our knowledge and according to recent systematic reviews, the relationship between SF and peak loading remains equivocal (Anderson et al., 2022; Schubert et al., 2014). Morin and colleagues (2007) state that SF exerts an indirect influence on external forces through its effect on contact time. However, the findings of the present study fail to establish a significant relationship between SF and any of the peak loading metrics. The latter is not unexpected taking the spring-mass dynamics into account. The spring-mass dynamics clearly demonstrate an inverse relationship of maximal vertical GRF, peak joint reaction forces and peak extensors moments at the ankle and the knee with the DF. However, a relationship with SF that is independent from the DF is absent (Bobbert and Casius, 2011; Bullimore and Burn, 2007).

In contrast to our hypotheses, SF did not correlate with the VILR. While PBF and FzMax are explained based on spring-mass dynamics, Bobbert and colleagues (1991) suggest that the VILR relates to the rapid deceleration of the mass of the foot and the shank as it strikes the ground. According to the two-mass model of Clark and colleagues (2017), lower magnitude of the VILR can be achieved by decreasing the deceleration of the lower limb during the impact phase or by slower deceleration of the remaining proximal parts during the running cycle. Interindividual differences in anthropometrics might explain why running with high SF does not necessarily result in a lower deceleration of the lower limb and as such, a VILR.

Although high DF runners experience less external loads, measures based on external ground reaction forces are a surrogate measure for internal forces. However, the DF shows an inverse relationship with peak extension moments and joint reaction forces at the knee and the ankle. The relationship between the DF and joint reaction forces is as strong as the relationship between the DF and FzMax. Our results, in combination with previous findings (Bonnaerens et al., 2019), show that recreational slow runners who run with high DFs experience less external and internal loads compared to recreational runners running with low DFs. In contrast, there is no relationship between running with high SF and lower external and internal loads. Since loading magnitude partly determines the development of RRIs, the relationship between the DF and loading magnitude can be transferred to the development of RRIs. Indeed, running with low DFs has recently been reported as a major risk factor for the development of RRIs (Malisoux et al., 2022), while running with low SF has not. As such, when the loading capacity of runners is assumed equal, a recreational slow runner running with high DFs experiences lower loading magnitude and may therefore be less prone for the development of RRIs, while high SF runners do not encounter these benefits.

In this study, we examined the habitual running style of female recreational runners without any deliberate alterations in the DF or SF. Our results indicate that recreational runners running with high DFs experience lower external and internal loads compared to low DF runners. As such, running coaches with a focus on promoting health-related benefits and running engagement should perhaps stop the promotion of short ground contact times and long flight times and focus on longer ground contact times and shorter flight times. Indeed, strong evidence regarding deliberate increases in the duty factor showed that for each increase in the DF with 1%, joint contact and peak muscle forces decrease up to 2.50% (Bonnaerens, 2022). However, the running style is spatiotemporally also defined by stride frequency. Many coaches and clinicians still believe that its increase is a good strategy in the management of RRIs despite the ambiguous results reported in literature. Limited evidence suggests that increasing the step rate by 5 to 30% does not alter FzMax and the VILR (Gabbett, 2020; Melcher et al., 2017; Pedisic et al., 2020b), while a single study found that in-field gait retraining in runners with high impact forces effectively reduced the VILR (Willy et al., 2016). As such, a strong within-subject study that alters the DF and SF is needed to examine the effect of a deliberate increase in the DF, SF and their interaction on the reduction of loading magnitude and eventually the prevention of RRIs.

A potential limitation of this study is the exclusive use of female recreational runners that ran from 3 to 30 km a week and had no injury three months prior to the study. This could lead to an overrepresentation of individuals who were less prone to injuries or who had adapted their running habits to prevent injuries, leading to stronger correlations between the DF, SF and loading measures in this group. Future research should focus more on novice runners or competitive athletes as these are at higher risk of the development of RRIs compared to recreational runners. Secondly, to guide gait retraining to prevent RRIs, prospective intervention studies in which both SF and the DF are altered within a participant should be performed.

## Conclusions

This study shows that runners who naturally run with higher duty factors experience lower external forces and measures derived from inverse dynamics compared to low duty factor runners. In contrast, there is no relationship between stride frequency and these measures as well as between stride frequency and the duty factor. These findings suggest that runners should focus on increasing the duty factor instead of stride frequency in order to reduce loading magnitude.
